# Supplementary figures and images for: Identification and Functional Validation of Auxin-Responsive Tabzip Genes from Wheat Leaves in Arabidopsis
Source: Int J Mol Sci. 2023 Jan 1;24(1):756. doi: 10.3390/ijms24010756 (PMC9821592; doi:10.3390/ijms24010756)

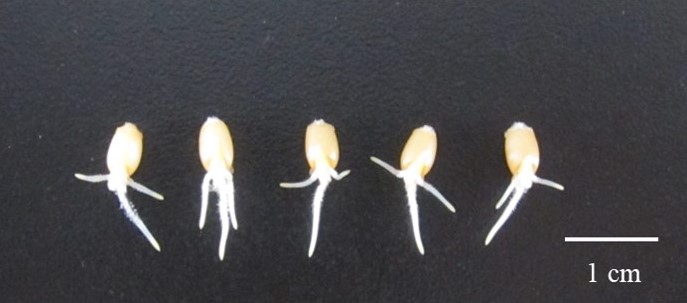

Supplement: Supplementary file 1 [file ijms-24-00756-s001.zip › Figure S1 Wheat seedling.jpg]

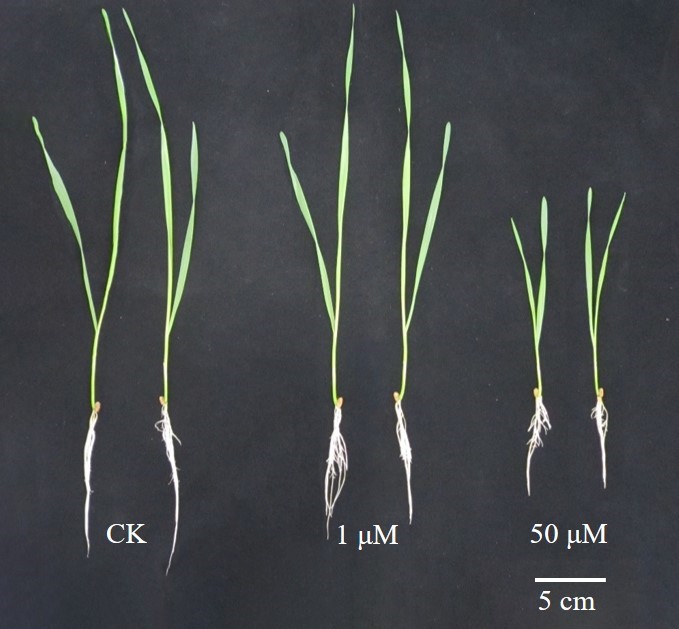

Supplement: Supplementary file 1 [file ijms-24-00756-s001.zip › Figure S2 Wheat seedling 1.jpg]

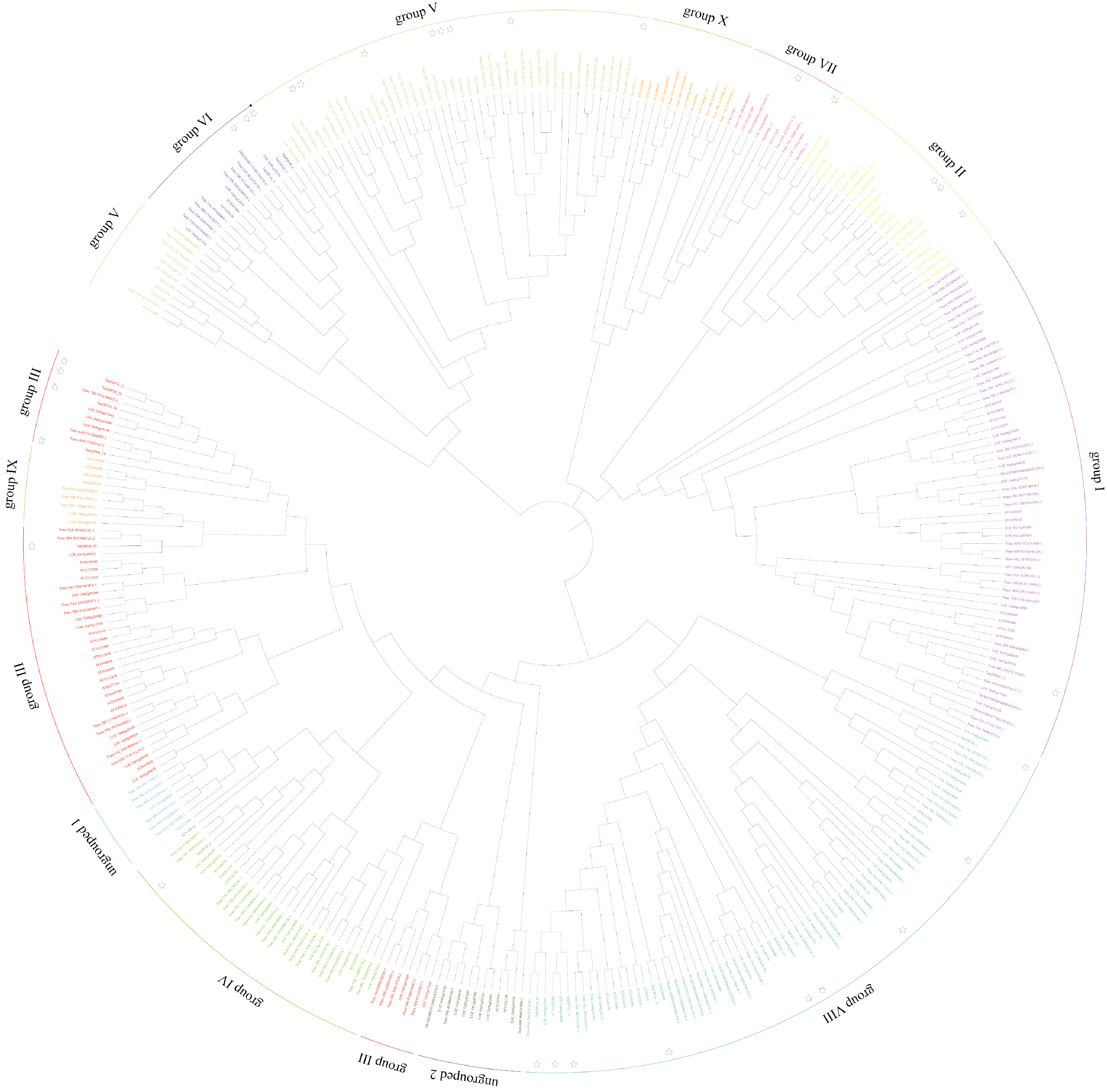

Supplement: Supplementary file 1 [file ijms-24-00756-s001.zip › Figure S3 Phylogenetic analysis of AT,TA and OS bZIP.png]

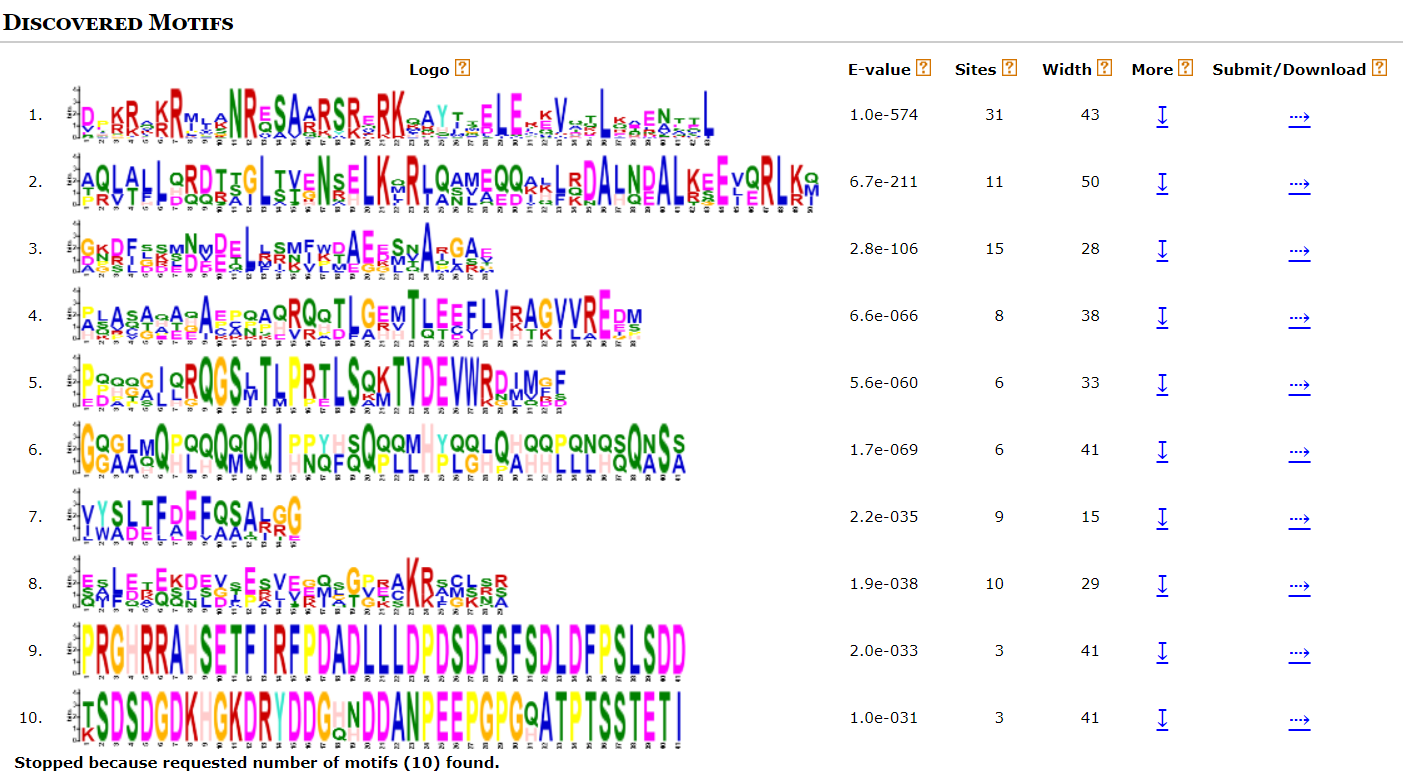

Supplement: Supplementary file 1 [file ijms-24-00756-s001.zip › Figure S4 Composition of motif.png]

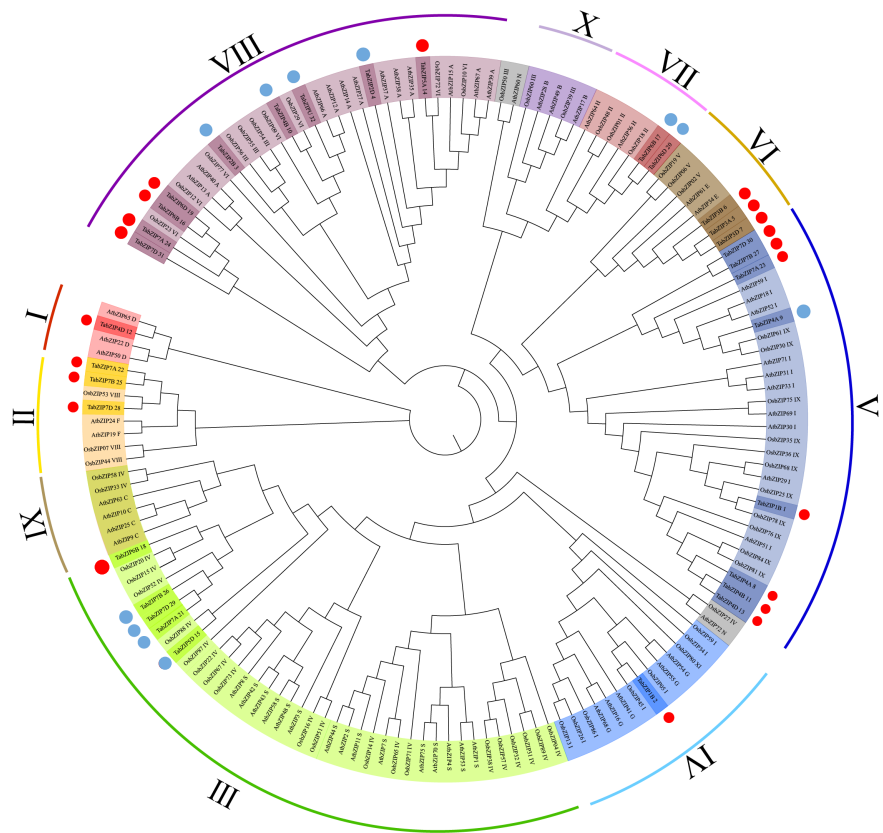

Supplement: Supplementary file 1 [file ijms-24-00756-s001.zip › Figure S5 Phylogenetic analysis of bZIP genes from T. Aestivum, Arabidopsis and O. sativa. High quality version.pdf]
